# Supplementary material for: Pressure-controlled magnetism in 2D molecular layers
Source: Nat Commun. 2023 Jun 2;14:3186. doi: 10.1038/s41467-023-38991-8 (PMC10238535; doi:10.1038/s41467-023-38991-8)
Supplement: Supplementary file 1 — Supporting Information [file 41467_2023_38991_MOESM1_ESM.pdf]

Supplementary Information

Pressure-Controlled Magnetism in 2D Molecular Layers

Yulong Huang[1,*], Arjun K. Pathak[2,*], Jeng-Yuan Tsai[3], Clayton Rumsey[4], Mathew Ivill[5], Noah Kramer[2], Yong Hu[1], Martin Trebbin[4,6], Qimin Yan[3,*], Shenqiang Ren[1,3,4,6,*]

[1]Department of Mechanical and Aerospace Engineering, University at Buffalo, The State University of New York, Buffalo, NY, 14260, USA

[2]Department of Physics, SUNY Buffalo State, Buffalo, New York 14222, USA

[3]Department of Physics, Northeastern University, Boston, MA 02115, USA

[4]Department of Chemistry, University at Buffalo, The State University of New York, Buffalo, NY, 14260, USA

[5]DEVCOM Army Research Laboratory, Aberdeen Proving Ground, MD 21005, USA

[6]Research and Education in Energy, Environment and Water (RENEW) Institute, University at Buffalo, The State University of New York, Buffalo, NY, 14260, USA

*E-mail: yhuang59@buffalo.edu; pathakak@buffalostate.edu; q.yan@northeastern.edu; shenren@buffalo.edu

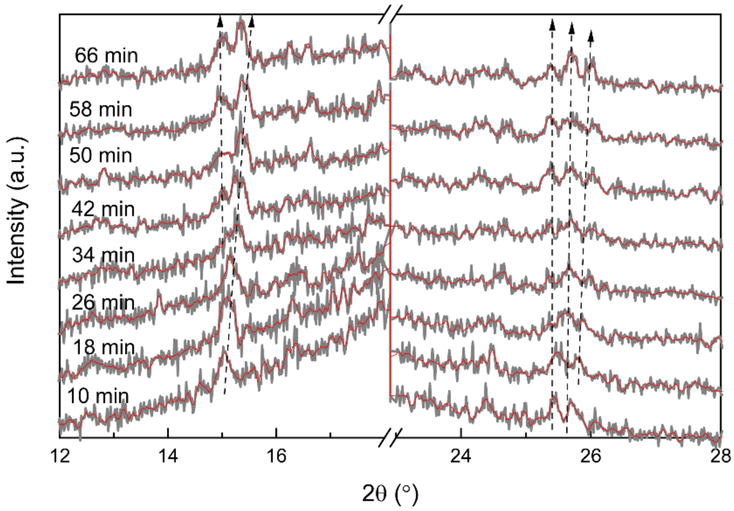

Figure S1. The X-ray diffraction reveals the in-situ structural change during the reaction between lithium solution and precursor $Cr(pyz)_2Cl_2$ for LCPC magnet. The peak shift and emergence around 15° and 26° show time-dependent structural evolution.

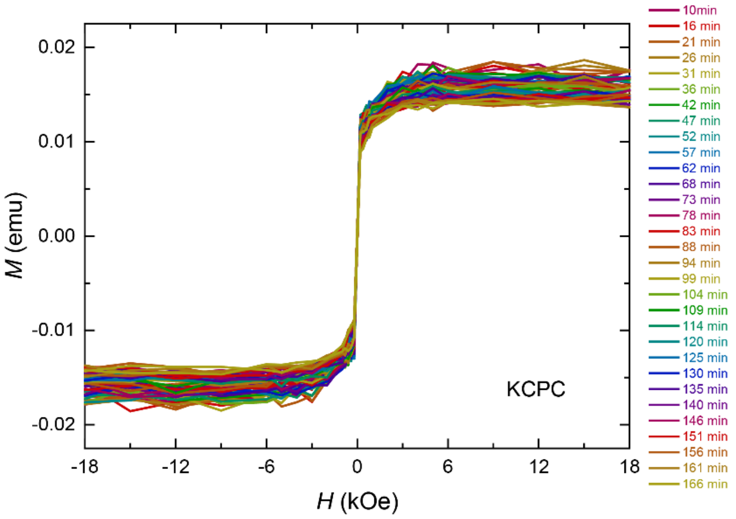

Figure S2. Magnetic hysteresis loops of KCPC magnet in-situ measured during synthesis. The soft magnetic behavior persists for the whole measurements.

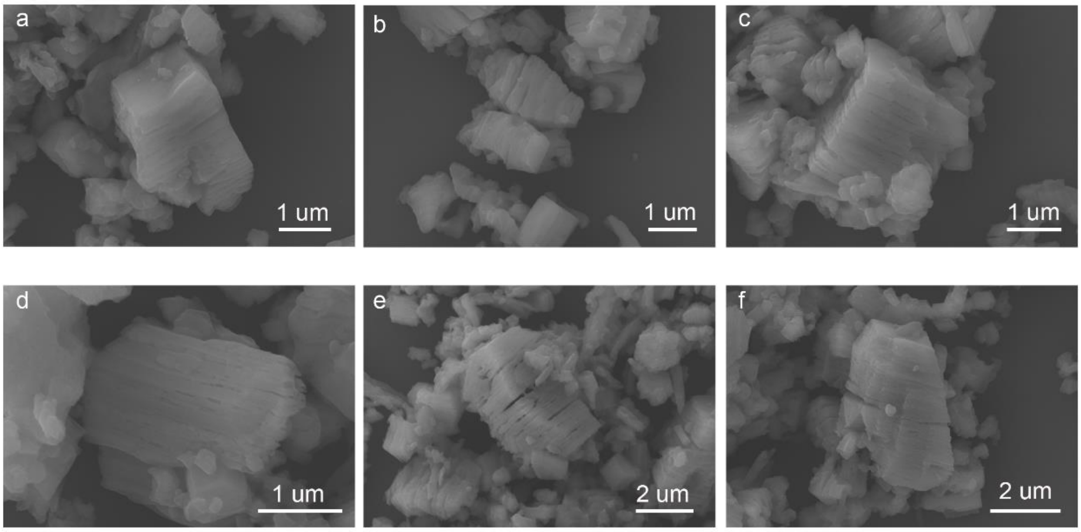

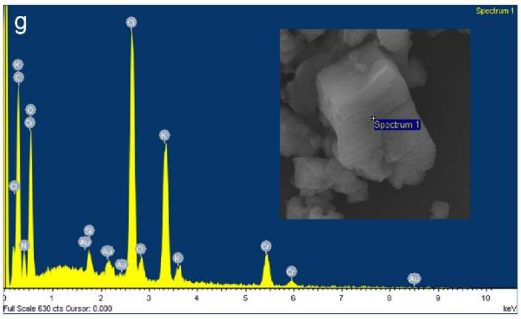

Element	Weight%	Atomic%
C K	22.48	40.21
N K	8.66	13.29
O K	13.50	18.14
Si K	0.79	0.60
Cl K	19.47	11.80
K K	16.70	9.18
Cr K	15.72	6.50
Au M	2.69	0.29

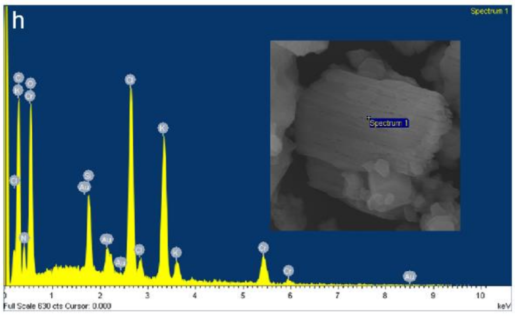

Element	Weight%	Atomic%
C K	20.27	36.71
N K	8.87	13.78
O K	16.05	21.83
Si K	3.04	2.35
Cl K	15.45	9.48
K K	16.93	9.42
Cr K	13.92	5.82
Au M	5.45	0.60

Figure S3. Morphology and element characterization of KCPC magnet. (a-f) Scanning electron microscopic images show the layered stacked structure. (g-h) Energy dispersive spectra (EDS) indicate all the elements (K, Cr, C, N, Cl, O) in LCPC magnet. Si element is from the silicon substrate; Au element is from sputtering deposition for a better surface conductivity.

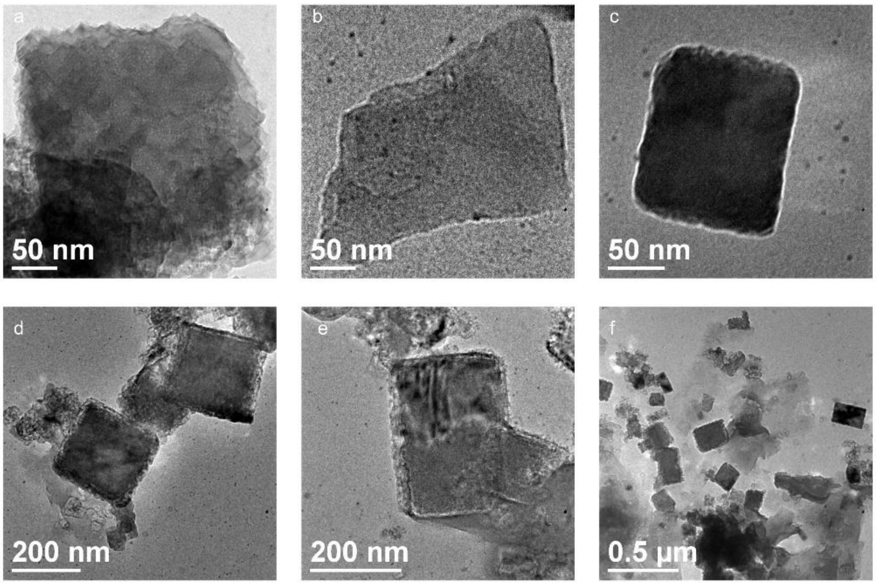

Figure S4. Transmission electron microscopy (TEM) images of KCPC magnet. (a-f) All the TEM images show the 2D layered feature.

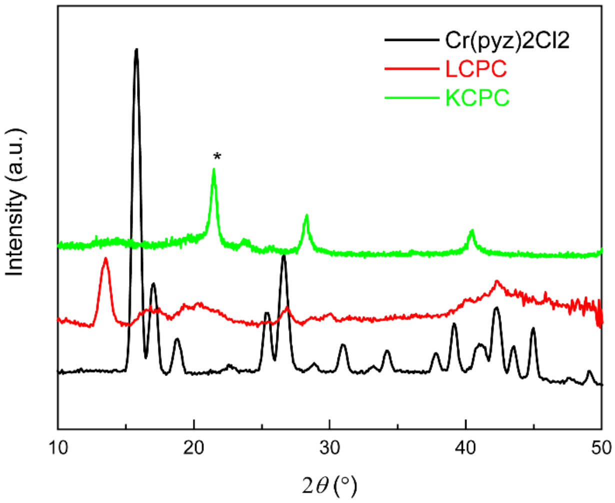

Figure S5. X-ray study of precursor $Cr(pyz)_2Cl_2$, LCPC and KCPC magnets. The wide-angle X-ray scattering patterns of precursor $Cr(pyz)_2Cl_2$ and LCPC indicate the structure transformation. The X-ray diffraction pattern of KCPC magnet also shows the structural difference. The peak marked by a star is from the protection layer on sample from oxidation.

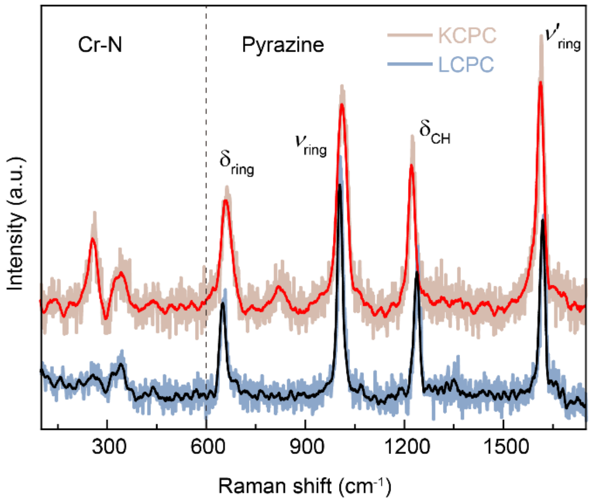

Figure S6. Raman spectra of LCPC and KCPC magnets. The Raman peaks below 600 cm^{-1} are assigned as Cr-N vibration modes, while internal vibration modes of pyrazine molecule appear from 600 cm^{-1} to 1700 cm^{-1}.

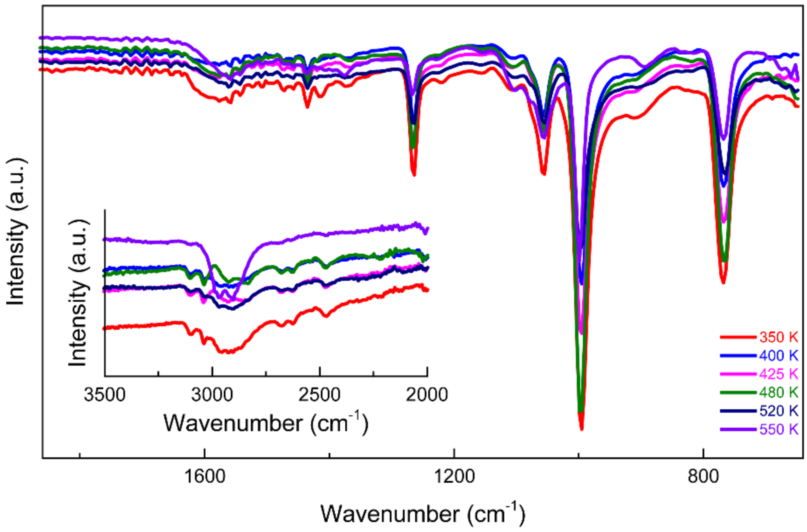

Figure S7. Fourier-transform infrared spectra of KCPC magnets annealed at different temperatures.

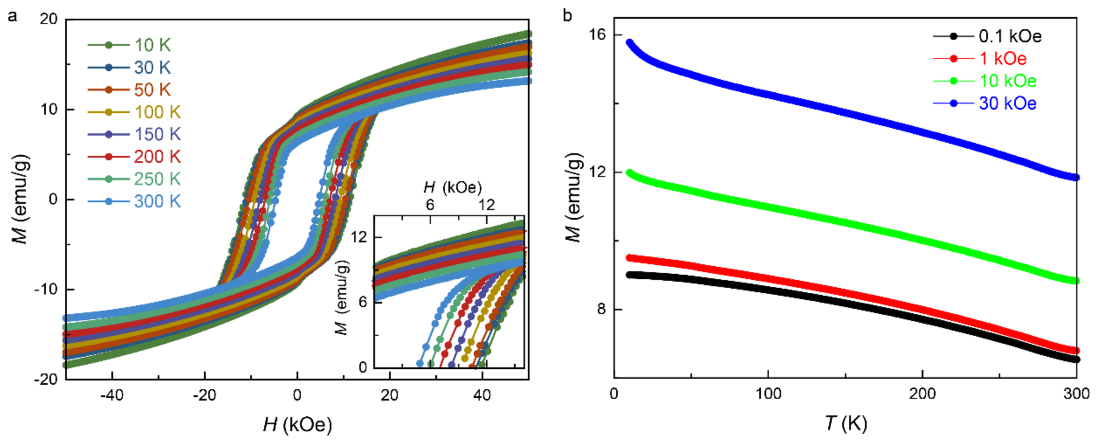

Figure S8. Magnetic properties of KCPC magnet #2. (a) Magnetic field dependent hysteresis loops were measured at different temperatures from 50 kOe to -50 kOe. (b) Low-temperature magnetization from 10 K to 300 K was measured at magnetic field of 0.1, 1, 10, and 30 kOe.

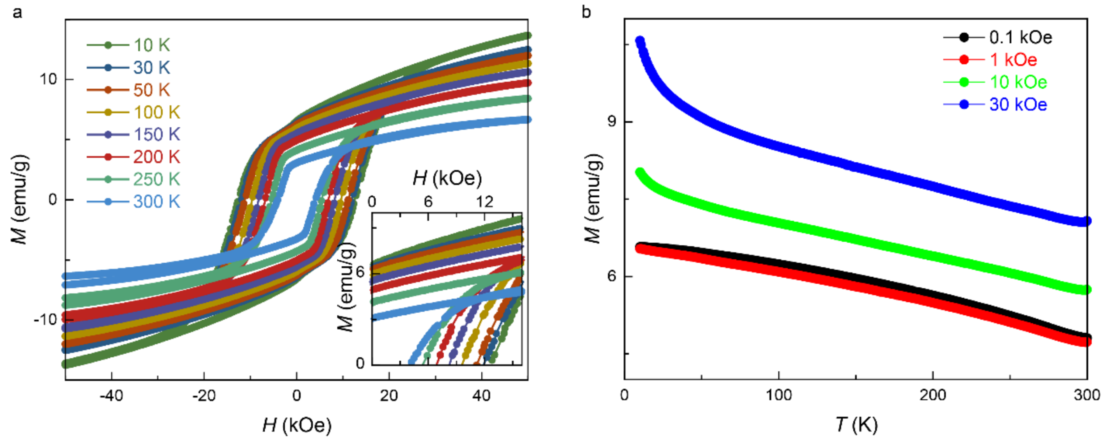

Figure S9. Magnetic properties of KCPC magnet #3. Magnetic field dependent hysteresis loops were measured at different temperatures from 50 kOe to -50 kOe. (b) Low-temperature magnetization from 10 K to 300 K was measured at magnetic field of 0.1, 1, 10, and 30 kOe.

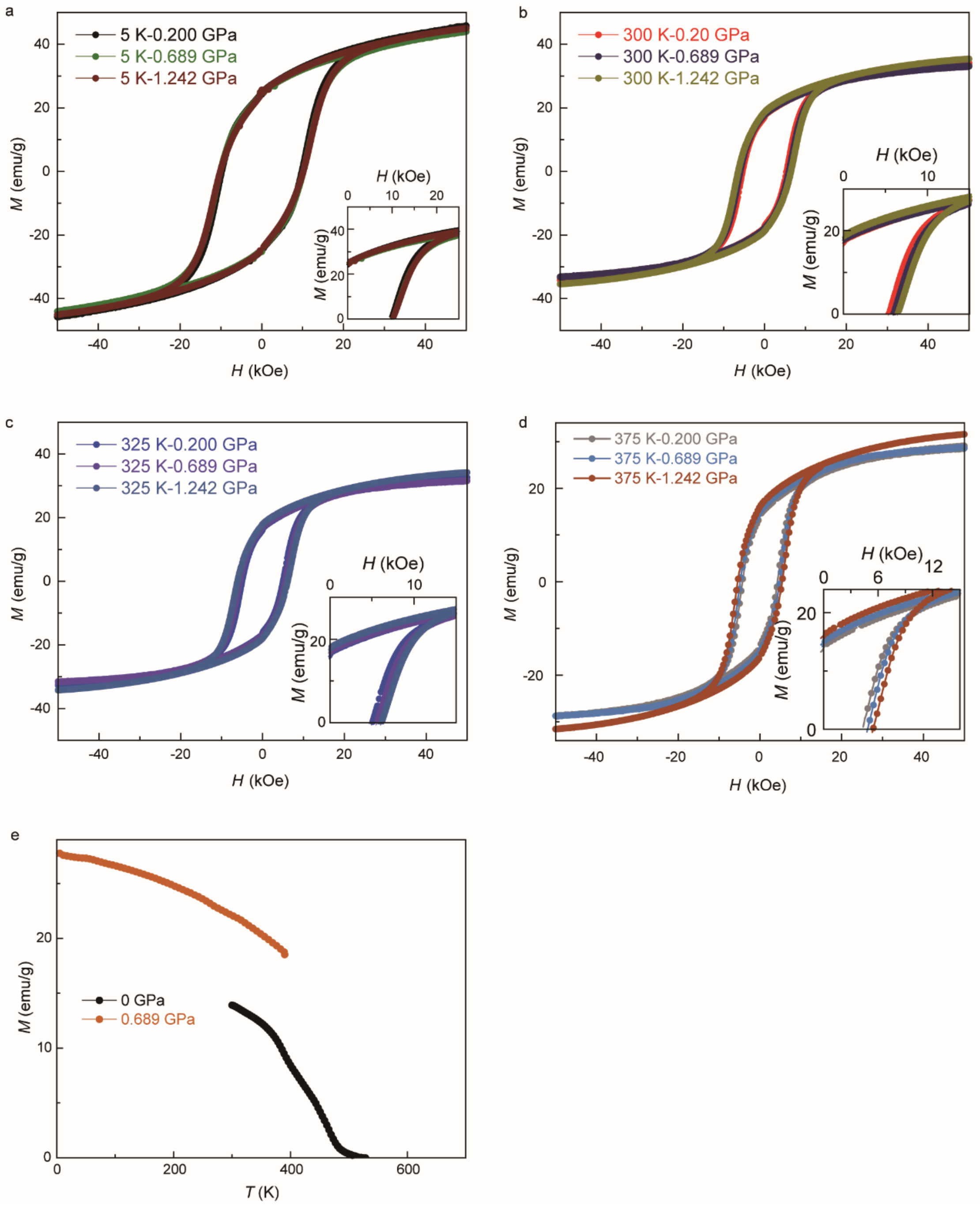

Figure S10. Pressure effect on magnetic properties of LCPC magnet. Magnetic hysteresis loops indicate pressure effect at (a) 5 K; (b) 300 K; (c) 325 K; (d) 375 K. The hydrostatic pressure increases from 0.200 GPa to 1.242 GPa. (e) Comparison of temperature-dependent magnetization measured at 0 and 0.689 GPa indicates the large enhancement on magnetization and magnetic ordering temperature.

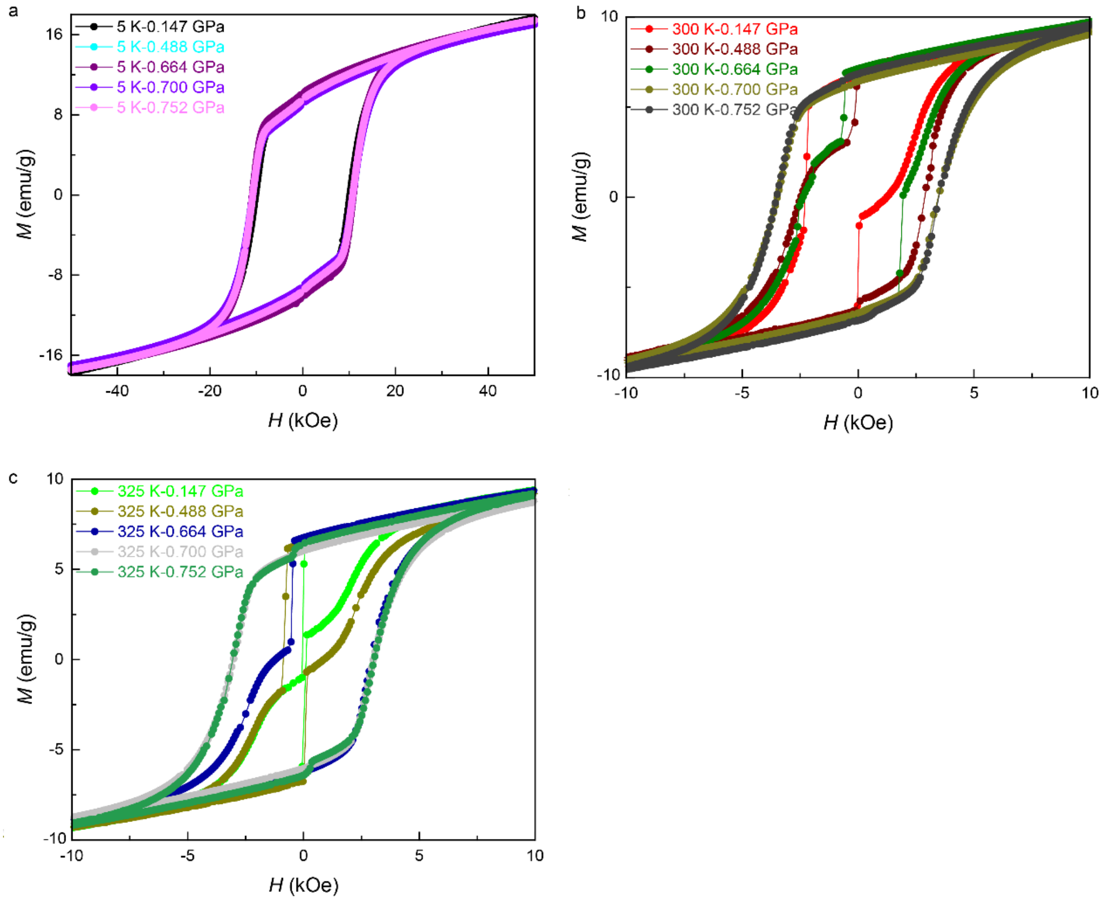

Figure S11. Pressure effect on magnetic properties of KCPC magnet. Magnetic hysteresis loops indicate pressure effect at (a) 5 K; (b) 300 K; (c) 325 K. The hydrostatic pressure increases from 0.147 GPa to 0.752 GPa.

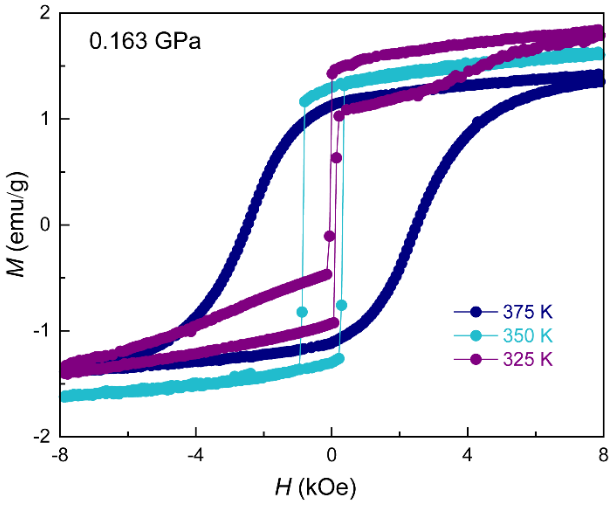

Figure S12. M-H loops of KCPC sample under 0.163 GPa.

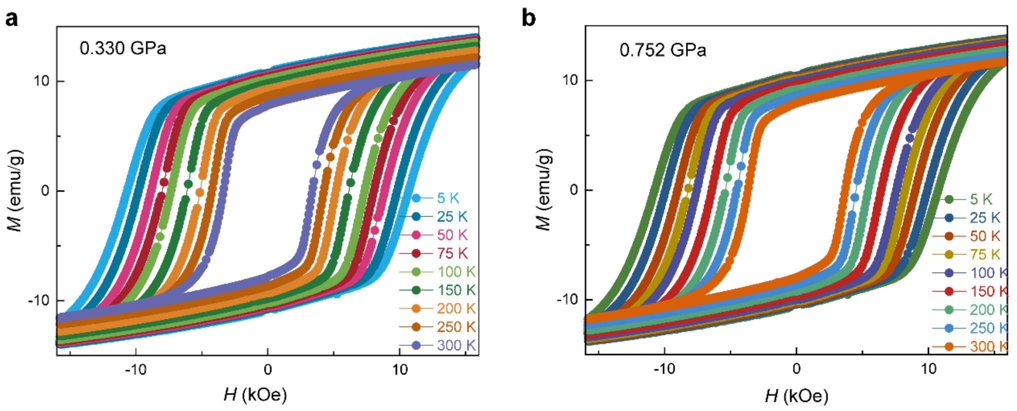

Figure S13. Low-temperature M-H loops of KCPC sample measured at (a) 0.330 GPa and (b) 0.752 GPa.

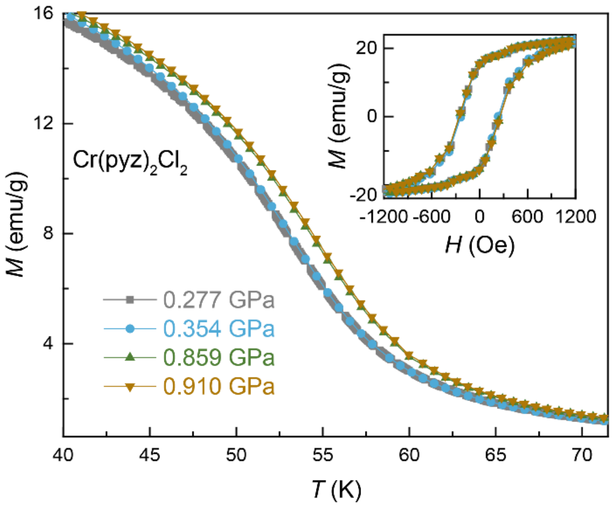

Figure S14. Pressure effect on magnetic properties of precursor $Cr(pyz)_2Cl_2$. Pressure increases the magnetic order transition temperature. The inset shows the magnetic hysteresis loops measured under different pressures at 5 K. No obvious changes are observed.

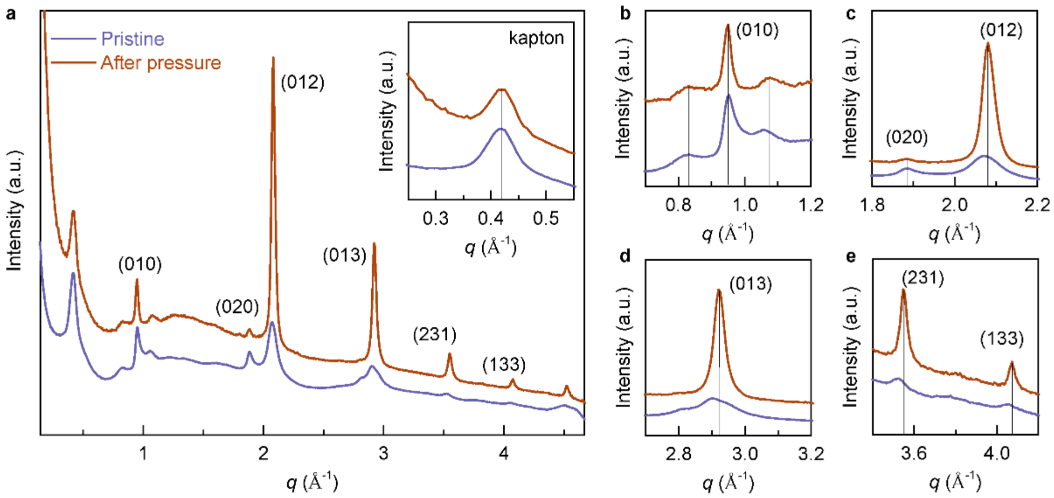

Figure S15. Wide-angle X-ray scattering (WAXS) patterns of KCPC samples before and after pressure. (a) The whole profiles of WAXS patterns show sharper and stronger peaks after pressure. (b-e) Peaks comparison indicates the shift to higher scattering vector.

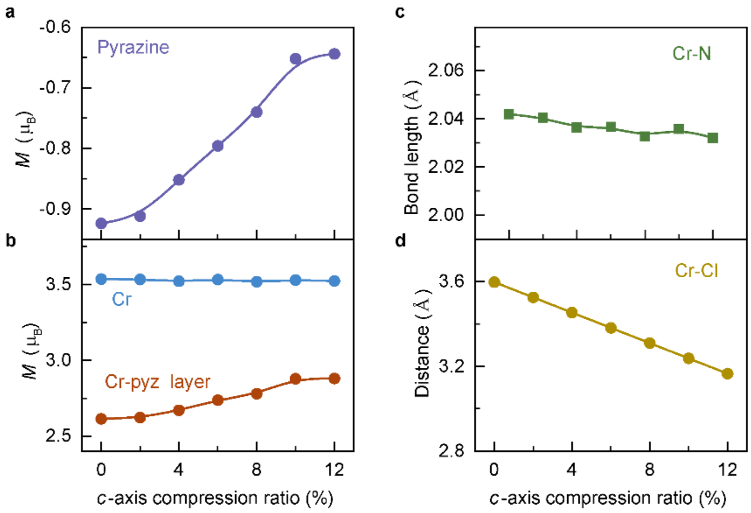

Figure S16. First-principles simulation of magnetism and structural parameters of KCPC with c-axis compression ratio up to 12%. (a) The magnetic moment of pyrazine molecule decreases in value with a compressed c-axis constant. (b) The magnetic moment of Cr is almost unchanged, while magnetic moment of Cr-pyz layer increases continuously. (c) Bond length of Cr-N in pyrazine molecule decrease, as well as the distance of Cr---Cl distance.

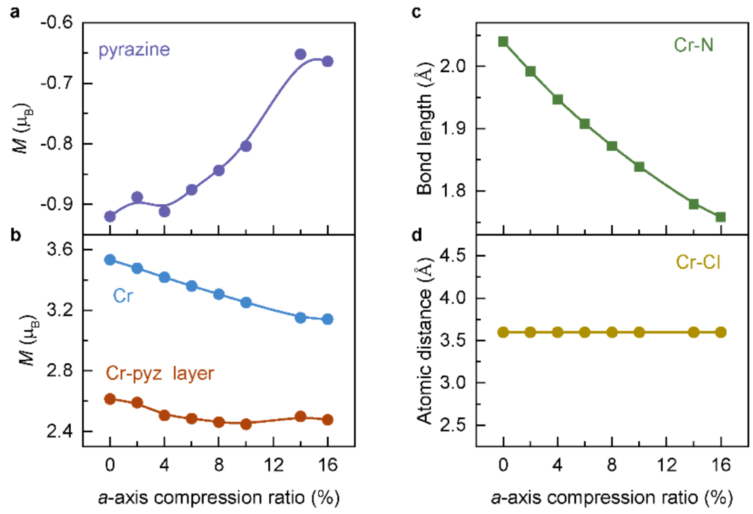

Figure S17. First-principles simulation of magnetism and structural parameters of KCPC with *a*-axis compression ratio up to 16%. (a) The magnetic moment of pyrazine molecule decreases in value with a compressed *a*-axis constant. (b) The magnetic moment of Cr continuously decreases with compression, while magnetic moment of Cr-pyz layer decreases first and then shows a little upturn. (c) Bond length of Cr-N in pyrazine molecule decrease, while Cr---Cl distance is kept unchanged as *c*-axis is not compressed.

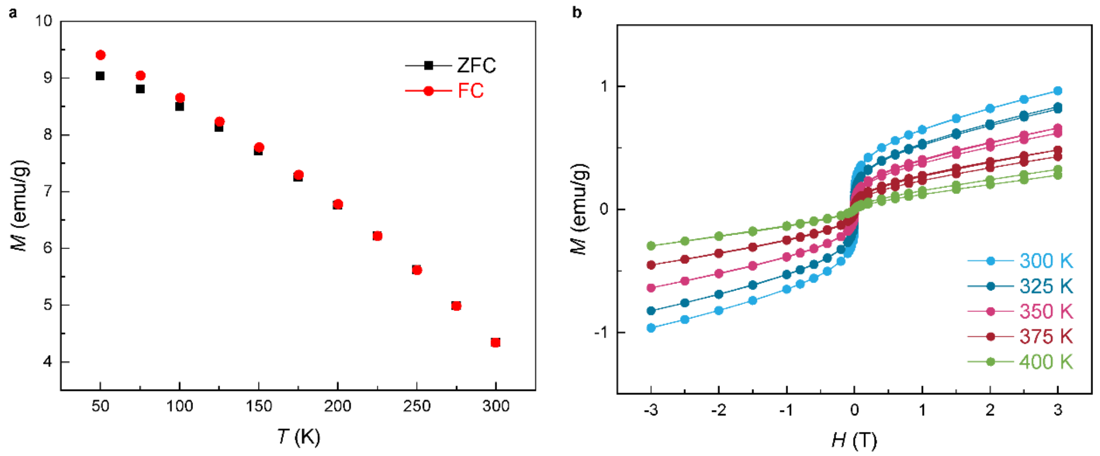

Figure S18. Magnetic properties of KCPC magnet sealed in epoxy after days still show room-temperature magnetism. (a) Temperature dependent magnetization was measured after 23 days. (b) Magnetic field dependent magnetization was measured after 50 days.
